# Streptolysin S induces pronounced calcium-ion influx-dependent expression of immediate early genes encoding transcription factors

**DOI:** 10.1038/s41598-023-40981-1

**Published:** 2023-08-22

**Authors:** Takuya Yamada, Yugo Yamamori, Nanami Matsuda, Hideaki Nagamune, Kazuto Ohkura, Toshifumi Tomoyasu, Atsushi Tabata

**Affiliations:** 1 Department of Biological Science and Technology, Life System, Institute of Technology and Science, Tokushima University Graduate School, 2-1 Minamijousanjima-Cho, Tokushima, Tokushima 770-8506 Japan; 2 Faculty of Bioscience and Bioindustry, Bioengineering Course, Tokushima University, 2-1 Minamijousanjima-Cho, Tokushima, Tokushima 770-8513 Japan; 3 Department of Bioengineering, Division of Bioscience and Bioindustry, Graduate School of Technology, Industrial and Social Sciences, Tokushima University Graduate School, 2-1 Minamijousanjima-Cho, Tokushima, Tokushima 770-8513 Japan; 4 Division of Clinical Pharmacy and Pharmaceutical Sciences, Graduate School of Pharmaceutical Sciences, Suzuka University of Medical Science, 3500-3 Minamitamagaki-Cho, Suzuka, Mie 513-8670 Japan

**Keywords:** Cell biology, Microbiology, Diseases, Pathogenesis

## Abstract

Anginosus group streptococci (AGS) are opportunistic human pathogens of the oral cavity. The β-hemolytic subgroup of *Streptococcus anginosus* subsp. *anginosus* secretes streptolysin S (SLS) and exhibits not only hemolytic activity but also cytotoxicity toward cultured human cell lines. However, the detailed mechanism of action of SLS and the cellular responses of host cells have not yet been fully clarified. To determine the pathogenic potential of SLS-producing β-hemolytic *S. anginosus* subsp. *anginosus*, the SLS-dependent response induced in the human oral squamous cell carcinoma HSC-2 cells was investigated to determine the pathogenic potential of SLS-producing β-hemolytic *S. anginosus* subsp. *anginosus*. This study revealed that the Ca^2+^ influx and the expression of immediate early genes (IEGs) encoding transcription factors such as early growth responses (EGRs) and activator protein-1 (AP-1) were greatly increased in HSC-2 cells incubated with the culture supernatant of SLS-producing β-hemolytic *S. anginosus* subsp. *anginosus*. Moreover, this SLS-dependent increase in expression was significantly suppressed by Ca^2+^ chelation, except for *jun*. These results suggest that SLS caused Ca^2+^ influx into the cells following greatly enhanced expression of IEG-encoding transcription factors. The results of this study may help in understanding the pathogenicity of SLS-producing AGS.

## Introduction

Streptolysin S (SLS) is a β-hemolysin produced by strains belonging to the pyogenic and anginosus groups of β-hemolytic streptococci^1,2^. SLS is generated as the product of an operon composed of 9 genes (*sagA*–*sagI*) called the “*sag* operon”^3^, except for the *sag* operon of β-hemolytic *S. anginosus* subsp. *anginosus*, which is composed of 10 genes (*sagA1*, *sagA2*, and *sagB*–*sagI*)^4,5^. The formation of heterocycle (thiazole and oxazole) rings in the precursor peptide is essential for the hemolytic activity of SLS. Thus, SLS is one of the bacteriocins called “thiazole and oxazole modified microcins (TOMMs)”^6^. The SLS has mainly been investigated in the human pathogenic *Streptococcus pyogenes*, and its contributions to hemolysis, cytotoxicity, and pathogenicity have been demonstrated both in vitro and in vivo^7–12^.

We are investigating the β-hemolytic factors produced by human opportunistic anginosus group streptococci (AGS). Among the species belonging to AGS, *Streptococcus intermedius* produces a cholesterol-dependent cytolysin (CDC) called intermedilysin (ILY) which is the sole β-hemolytic factor of the species with human cell-specific action^13,14^. In contrast, the β-hemolytic subgroup of other AGS species, such as *S. anginosus* and *S. constellatus*, produce the homologs of SLS as the sole β-hemolytic factors^4,15^. Interestingly, β-hemolytic strains of *S. anginosus* subsp. *anginosus* produce two functional mature SLSs with different amino acids^4^. Moreover, the SLS secreted by β-hemolytic AGS strains shows not only hemolytic activity but also cytotoxicity^15,16^. Recently, we reported that SLS secreted by hemolytic AGS strains and other SLS-producing streptococci was stabilized in the presence of human serum albumin (HSA) and showed enhanced hemolysis and cytotoxicity^17^. Based on this information, we suggested that the presence of gingival crevicular fluid (GCF) is suitable for the growth of SLS-producing AGS strains that exhibit SLS-dependent hemolytic activity and cytotoxicity. In lesions such as gingivitis and periodontal disease, the secreted SLS is apparently stabilized in the presence of HSA and may contribute to disorders in the oral cavity as well as in other parts where the β-hemolytic AGS is observed ectopically. However, the exact mechanism of the action of SLS secreted by β-hemolytic AGS on host cells and the cellular responses to SLS remain unclear.

In the present study, to determine the detailed mechanism of action of SLS secreted by β-hemolytic AGS strains, the HSA-stabilized SLS-dependent cellular response in the human oral squamous cell carcinoma cell line HSC-2 was investigated, focusing on the effect on gene expression.

## Results

### SLS-dependent cytotoxicity against HSC-2 cells

**Figure 1:**
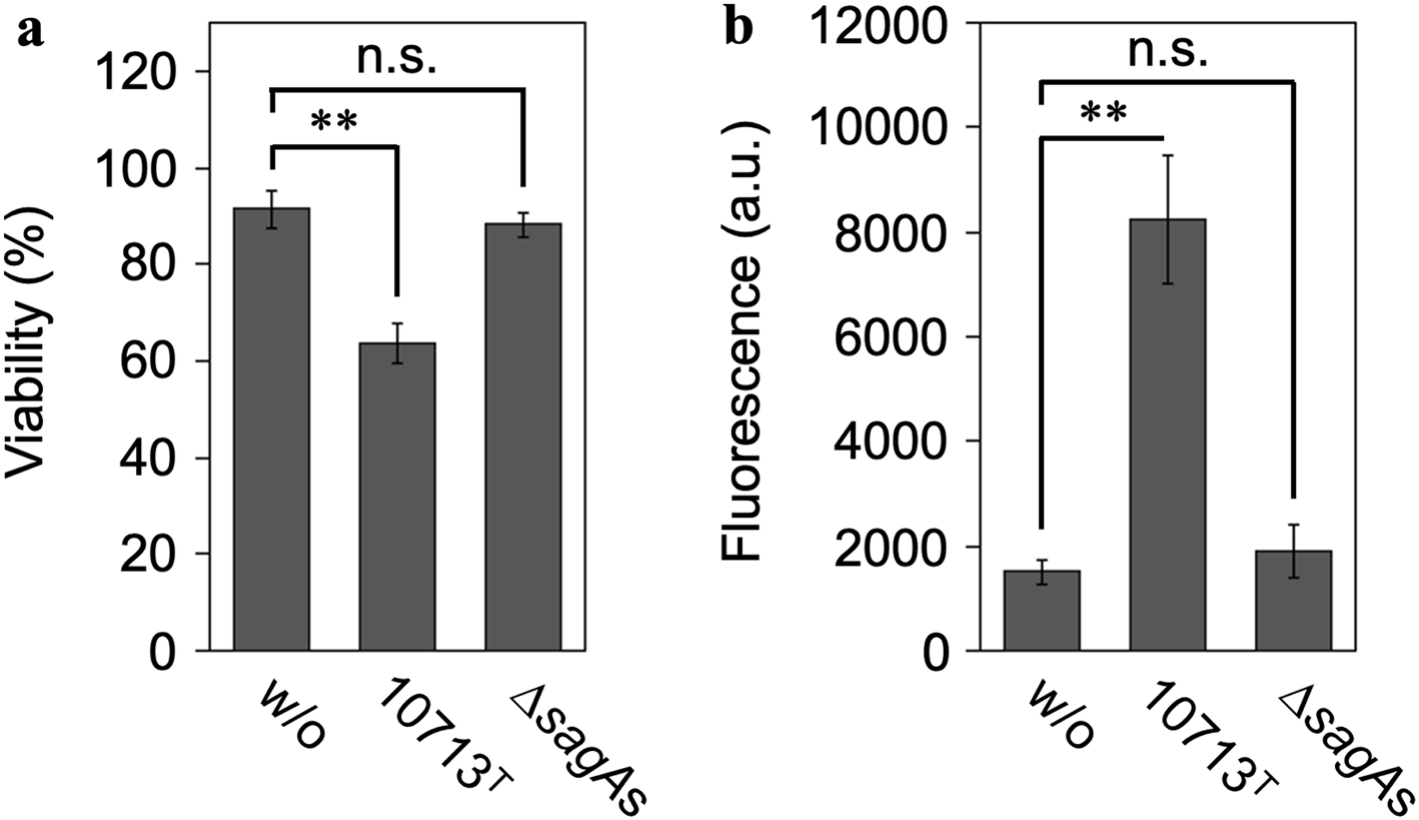
SLS-dependent cytotoxicity toward the human oral squamous cell carcinoma cell line HSC-2. The viability of HSC-2 cells incubated with the culture supernatant prepared from *S. anginosus* subsp. *anginosus* strain NCTC10713^T^ or its *sagA*-genes (*sagA1* and *sagA2*) deletion mutant (∆*sagA*s) was evaluated by CCK-8 assay (Dojindo) (**a**) and by CellTox™ Green assay (Promega) (**b**). The “w/o” indicates HSC-2 cells incubated with the co-cultivation medium as the control. Triplicate samples were assayed, and the result is presented as the mean ± SD. The significance of the SLS-dependent cytotoxicity was evaluated by Students’ *t*-test (**p < 0.01, *n.s.* not significant). *a.u.* arbitrary unit.

The SLS-dependent cytotoxicity toward HSC-2 cells in the culture supernatant of the tested strains was investigated. The Cell Counting Kit-8 (CCK-8; Dojindo, Masuki, Kumamoto, Japan) assay, which measures the cellular metabolic activity, revealed a partial but significant decrease in the viability of HSC-2 cells incubated with the culture supernatant of the β-hemolytic *S. anginosus* subsp. *anginosus* strain NCTC10713^T^. In contrast, no significant decrease in viability was observed in HSC-2 cells incubated with the culture supernatant of the non-β-hemolytic mutant strain Δ*sagA*s (Fig. 1a). In addition, the CellTox™ Green Cytotoxicity Assay (Promega, Madison, WI, USA), which reflects the intactness of the cell membrane, showed significant cytotoxicity in HSC-2 cells incubated with the culture supernatant of NCTC10713^T^ but not in those incubated with the culture supernatant of Δ*sagA*s (Fig. 1b). Therefore, it was confirmed that SLS secreted into the culture medium containing 1.0% (w/v) HSA could induce significant cytotoxicity via cell membrane damage and decrease the viability of HSC-2 cells.

### SLS-dependent Ca^2+^ influx into HSC-2 cells

**Figure 2:**
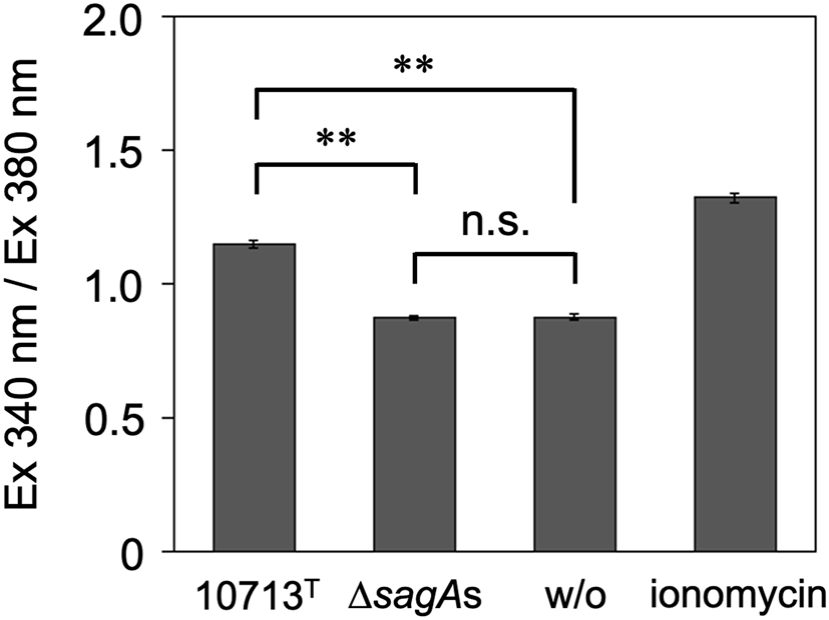
SLS-dependent influx of extracellular Ca^2+^ into HSC-2 cells. The influx of Ca^2+^ into HSC-2 cells incubated with the culture supernatant prepared from the tested strains was evaluated using Fura-2 Calcium Kit (Dojindo). HSC-2 cells treated with 0.1 μM ionomycin were used as the Ca^2+^-permeabilized control for this assay. The “w/o” indicates HSC-2 cells incubated with the co-cultivation medium as the control without cultivating the tested strains. Triplicate samples were assayed, and the result is presented as the mean ± SD. The significance of the SLS-dependent increase of Ca^2+^ influx was evaluated by Students’ *t*-test (**p < 0.01, *n.s.* not significant).

SLS-induced intracellular Ca^2+^ influx was investigated. A significant increase in intracellular Ca^2+^ was observed in HSC-2 cells incubated with the culture supernatant of NCTC10713^T^ (Fig. 2). In contrast, no such increase was observed in HSC-2 cells incubated with the culture supernatant of Δ*sagA*s, and the intracellular Ca^2+^ level was almost the same as that in HSC-2 cells incubated with the co-cultivation medium alone (w/o) (Fig. 2). These results suggest that SLS secreted by NCTC10713^T^ induces significant Ca^2+^ influx into HSC-2 cells.

### SLS-dependent gene expression in HSC-2 cells

**Figure 3:**
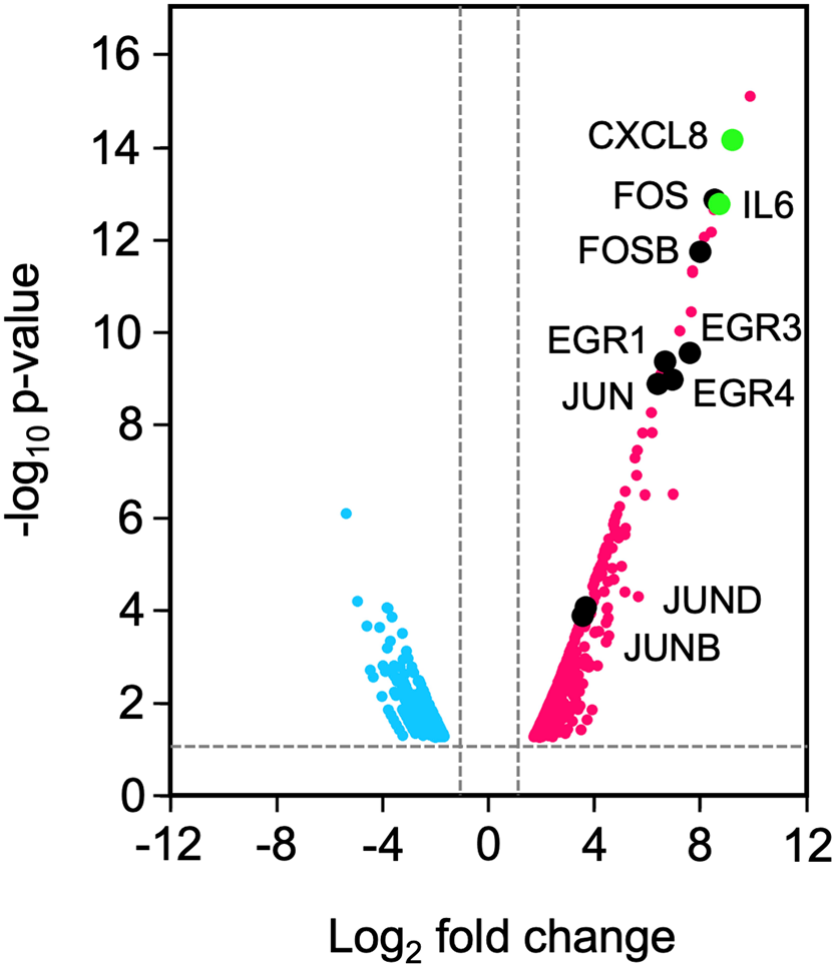
Volcano plot of the SLS-dependent induction (magenta) and reduction (cyan) of the gene expression in HSC-2 cells incubated with the culture supernatant prepared from *S. anginosus* subsp. *anginosus* strain NCTC10713^T^. The SLS-dependent induction or reduction of the expression of genes in HSC-2 cells was evaluated using the results of RNA-seq analysis outsourced to Macrogen Japan. The genes focused on in this study are indicated with black symbols (IEGs) and green symbols (cytokine genes) with the product name.

RNA-seq analysis was conducted to investigate SLS-dependent changes in gene expression. Differentially expressed genes (DEG) were analyzed in HSC-2 cells incubated with culture supernatants prepared from NCTC10713^T^ and Δ*sagA*s. The results are presented as a volcano plot showing the changes in gene expression (Fig. 3). SLS-dependent increases and decreases in expression were observed for 433 and 269 genes, respectively. Interestingly, the expression of the genes encoding transcription factors categorized as “immediate-early genes (IEGs)” and some cytokine-encoding genes was remarkably increased in HSC-2 cells incubated with the culture supernatant containing SLS. Among these, genes encoding early growth response (EGR), Fos family proteins, Jun family proteins, and inflammatory cytokines (IL-6 and CXCL8) were selected for further investigation.

### SLS-dependent IEG expression is significantly suppressed in HSC-2 cells by Ca^2+^ chelation

**Figure 4:**
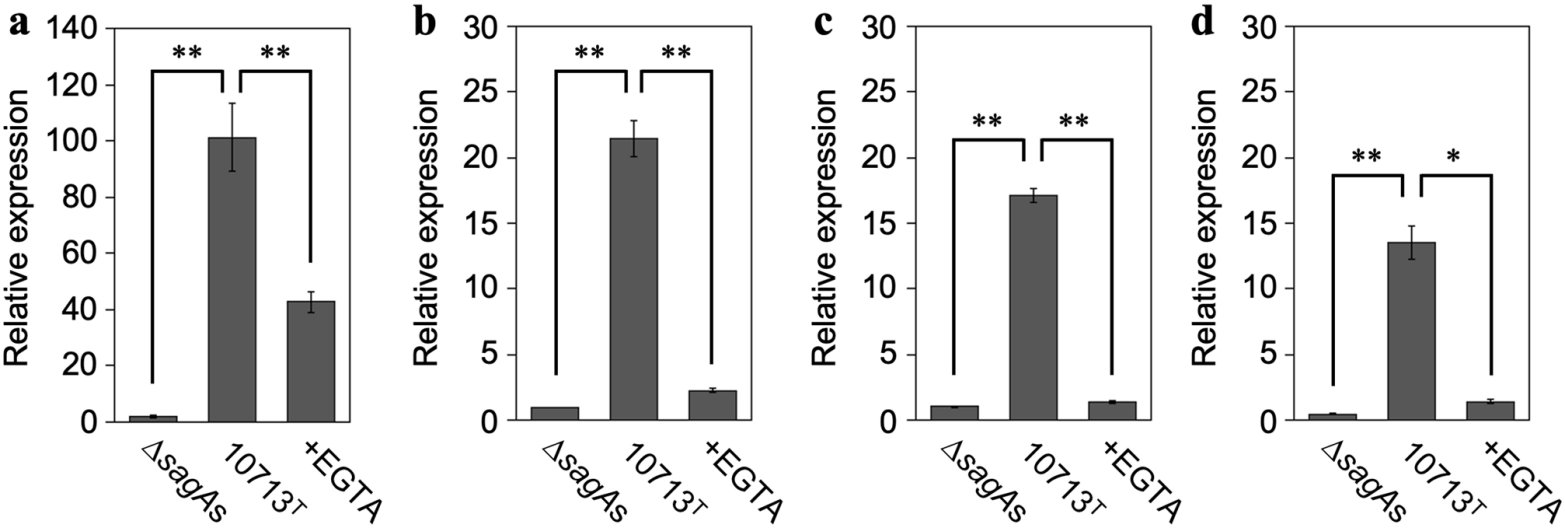
SLS-dependent and Ca^2+^-influx-induced expression of the *egr* genes, *egr1* (**a**), *egr2* (**b**), *egr3* (**c**), and *egr4* (**d**). HSC-2 cells were incubated with the culture supernatant prepared from *S. anginosus* subsp. *anginosus* strain NCTC10713^T^ or its *sagA*-genes (*sagA1* and *sagA2*) deletion mutant (∆*sagA*s). The contribution of Ca^2+^ influx to the expression of the genes was also evaluated in the presence of 5 mM EGTA. Triplicate samples were assayed, and the representative result is presented as the mean ± SD. These results were described as the relative expression against the control sample of HSC-2 cells incubated with co-cultivation medium. The significance of the increase of SLS-dependent and Ca^2+^-influx-dependent expression was evaluated by Welch’s or Students’ *t*-test (**p < 0.01, *p < 0.05).

The results of the DEG analysis (Fig. 3) showed increased expression of three *egr* genes (*egr1*, *egr3*, and *egr4*). As four *egr* genes (*egr1*, *egr2*, *egr3*, and *egr4*) have been reported to date, they were further investigated using quantitative real-time polymerase chain reaction (RT-qPCR) to reveal the relationship between SLS-dependent increases in the expression of IEGs and Ca^2+^ influx. The expression of all *egr* genes was greatly increased by incubation with culture supernatants containing SLS (Fig. 4a–d, 10713^T^ compared with ∆*sagA*s). The SLS-dependent increase in expression was significantly decreased by Ca^2+^ chelation (Fig. 4a–d, + EGTA compared with 10713^T^). These results showed that the SLS-dependent expression of all *egr* genes was triggered by Ca^2+^ influx, according to the SLS-dependent damage to the cellular membrane.

**Figure 5:**
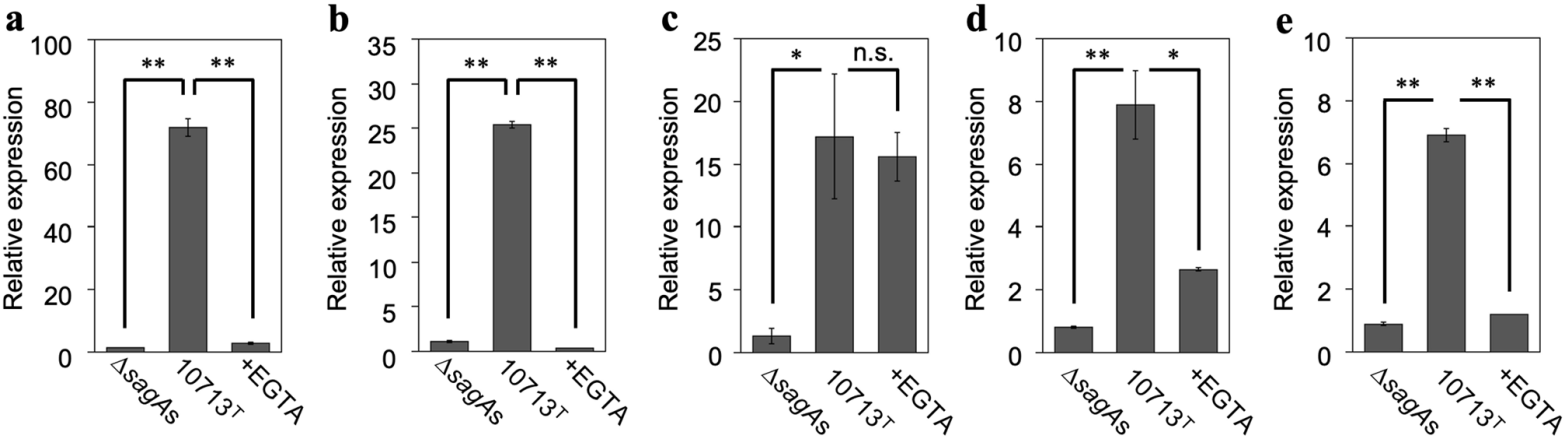
SLS-dependent and Ca^2+^-influx-induced expression of *fos* (**a**), *fosB* (**b**), *jun* (**c**), *junB* (**d**), and *junD* (**e**). HSC-2 cells were incubated with the culture supernatant prepared from *S. anginosus* subsp. *anginosus* strain NCTC10713^T^ or its *sagA*-genes (*sagA1* and *sagA2*) deletion mutant (∆*sagA*s). The contribution of Ca^2+^ influx to the expression of the genes was also evaluated in the presence of 5 mM EGTA. Triplicate samples were assayed, and the representative result is presented as the mean ± SD. These results were described as the relative expression against the control sample of HSC-2 cells incubated with co-cultivation medium. The significance of the increase of SLS-dependent and Ca^2+^-influx-dependent expression was evaluated by Welch’s or Students’ *t*-test (**p < 0.01, *p < 0.05, *n.s.* not significant).

Next, we investigated the SLS-dependent increase in *fos* gene expression. As shown in Fig. 5a and b, the expression of *fos* and *fosB* was greatly increased after incubation with the SLS-containing culture supernatant (10713^T^ compared with ∆*sagA*s). This SLS-dependent increase in expression was significantly suppressed to the background levels by Ca^2+^ chelation (Fig. 5a and b, + EGTA compared with 10713^T^). Thus, these results indicate that the SLS-dependent expression of *fos* genes was upregulated in a Ca^2+^-dependent manner, similar to that of *egr* genes.

The SLS-dependent expression of *jun* genes was also investigated. As shown in Fig. 5c–e, a significant increase in the expression of the three *jun* genes was observed after incubation with SLS-containing culture supernatant (10713^T^ compared with ∆*sagA*s), with a similar trend to that observed for the *egr* and *fos* genes. The expression of these genes is associated with Ca^2+^ influx since the SLS-dependent expression of *junB* and *junD* was significantly decreased by Ca^2+^ chelation (Fig. 5d and e, + EGTA compared with 10713^T^), as were the *egr* genes and *fos* genes. However, only the expression of *jun* gene under the Ca^2+^-chelating conditions was different (Fig. 5c, + EGTA). No significant change was observed in the SLS-dependent increase in the expression of *jun* even under Ca^2+^-chelating conditions (+ EGTA compared with 10713^T^). This interesting result suggests that the SLS-dependent pathway that induces *jun* expression differs from that of *junB* and *junD*.

### SLS-dependent increased expression of cytokine genes is also dependent on the Ca^2+^ influx in HSC-2 cells

**Figure 6:**
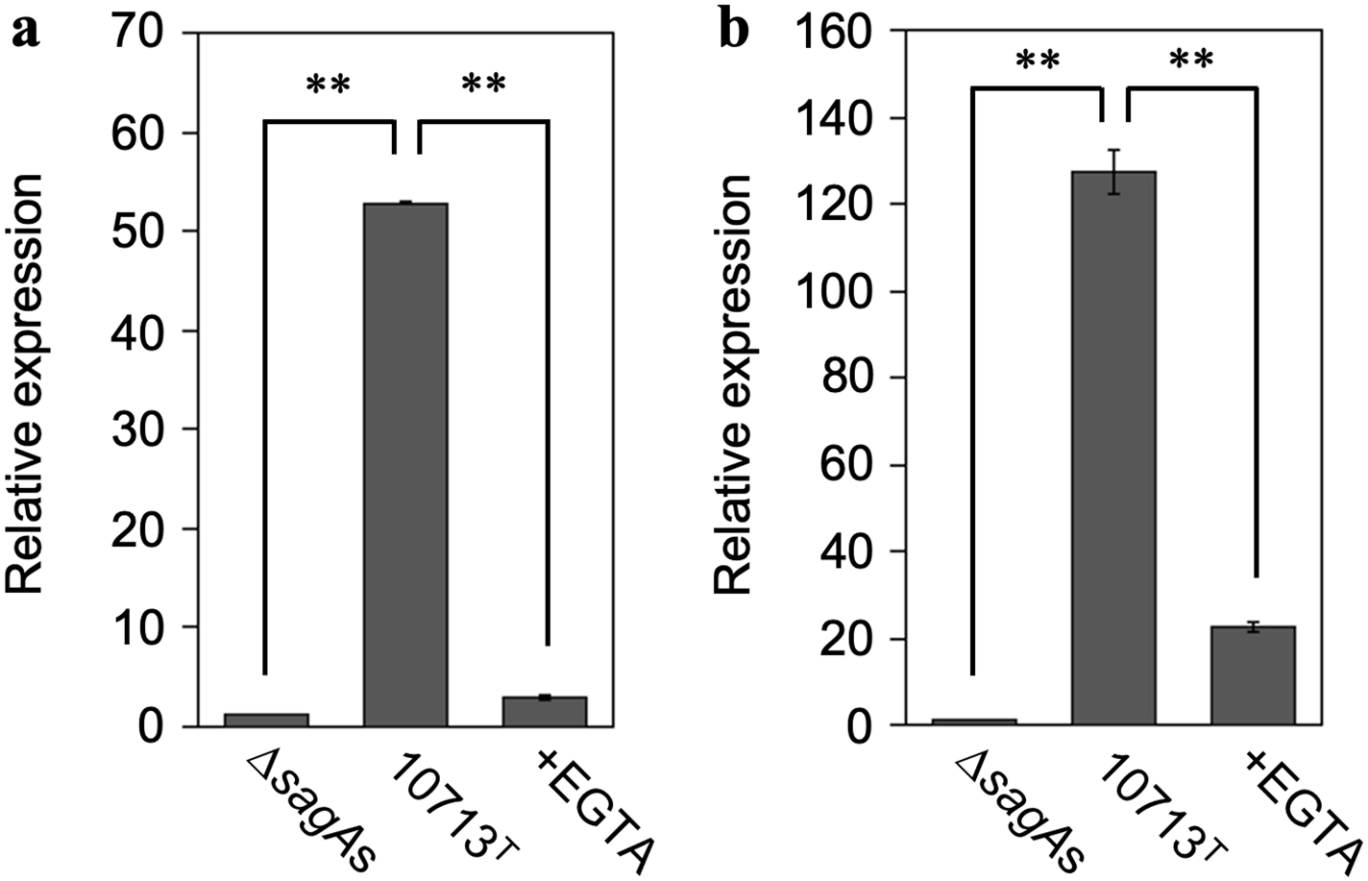
SLS-dependent and Ca^2+^-influx-induced expression of the genes encoding IL-6 (**a**) and CXCL8 (**b**). HSC-2 cells were incubated with the culture supernatant prepared from *S. anginosus* subsp. *anginosus* strain NCTC10713^T^ or its *sagA*-genes (*sagA1* and *sagA2*) deletion mutant (∆*sagA*s). The contribution of Ca^2+^ influx to the expression of the genes was also evaluated in the presence of 5 mM EGTA. Triplicate samples were assayed, and the representative result is presented as the mean ± SD. These results were described as the relative expression against the control sample of HSC-2 cells incubated with co-cultivation medium. The significance of the increase of SLS-dependent and Ca^2+^-influx-dependent expression was evaluated by Welch’s or Students’ *t*-test (**p < 0.01).

We also investigated whether the SLS-dependent increase in the expression of the inflammatory cytokine genes encoding IL-6 and CXCL8 (IL-8) depended on Ca^2+^ influx (Fig. 6). RT-qPCR analysis revealed that the expression of both genes was greatly increased in HSC-2 cells treated with culture supernatants containing SLS (10713^T^ compared with Δ*sagA*s), and this SLS-dependent expression was significantly decreased by Ca^2+^ chelation (+ EGTA compared with 10713^T^). These results strongly suggest that the SLS-dependent increase in the expression of the genes encoding IL-6 and CXCL8 in HSC-2 cells also depend on Ca^2+^-influx.

## Discussion

*S. anginosus* is an opportunistic pathogen that generally inhabits the human oral cavity. However, reports on the pathogenicity of this species in humans are increasing. For example, this species has been isolated from parts other than the oral cavity in cases of infection and other disorders^18^. Therefore, *S. anginosus* is currently attracting attention as a causative bacterium of ectopic infections in humans. There are only two subspecies of *S. anginosus*: subsp. *anginosus* and subsp. *whileyi*^19^. *S. anginosus* subsp. *whileyi* exhibits a β-hemolysis due to the production of SLS encoded by the *sag* operon which includes nine genes^15^. In contrast, both β-hemolytic and non-β-hemolytic strains are present in the *S. anginosus* subsp. *anginosus*. The hemolytic factor of β-hemolytic subgroup of *S. anginosus* subsp. *anginosus* consists of two SLSs with different amino acid sequences^4,5^. The amino acid sequence of each of SLS precursor (SagA1 and SagA2) was highly conserved among the strains of β-hemolytic *S. anginosus* subsp. *anginosus* (Table 1).

**Table 1:** Amino acid sequence identity and similarity of SLS precursor (SagA1 and SagA2) among sequenced strains of *S. anginosus* subsp. *anginosus*.

SAA strain (GenBank ID)	SagA1		SagA2	
	Identity	Similarity	Identity	Similarity
R87/1657 (JN619422.1)	54/54 (100%)	54/54 (100%)	50/50 (100%)	50/50 (100%)
R84/4972 (JN619421.1)	54/54 (100%)	54/54 (100%)	49/50 (98%)	50/50 (100%)
CCUG27928 (AP018548.1)	54/54 (100%)	54/54 (100%)	50/50 (100%)	50/50 (100%)
K20 (JAASHW010000002.1)	54/54 (100%)	54/54 (100%)	50/50 (100%)	50/50 (100%)
T5 (BASY01000013.1)	54/54 (100%)	54/54 (100%)	50/50 (100%)	50/50 (100%)
47S1 (CP088916.1)	54/54 (100%)	54/54 (100%)	50/50 (100%)	50/50 (100%)

SLS is a peptide hemolysin generally produced by pyogenic group streptococci such as *S. pyogenes*. In vitro studies have revealed that SLS contributes to epithelial barrier translocation^9^, programmed cell death and inflammatory signaling^10^, inflammation and cytotoxicity via NBCn1^12^, and mitochondrial damage and macrophage death by inhibition of GSK-3β degradation^20^. Moreover, in vivo investigations using mouse and zebrafish models^21^ have reported the recruitment of neutrophils at an early stage of infection. Based on the results of numerous studies, SLS produced by *S. pyogenes* is generally recognized as an important pathogenic factor in this species.

Although the cytotoxicity of SLS produced by *S. anginosus* has been previously reported^15,16,22^, the detailed mechanism of SLS-dependent cytotoxicity and the contribution of *S. anginosus*-produced SLS to human pathogenicity remain unclear. Recently, we reported that the cytotoxicity of SLS secreted from the β-hemolytic *S. anginosus* subsp. *anginosus* strain NCTC10713^T^ and other SLS-producing streptococci was significantly stabilized in the presence of HSA^17^. This property shows that β-hemolytic *S. anginosus* subsp. *anginosus* can exhibit cytotoxicity in the presence of serum albumin in, for example, the GCF and lesions of gingivitis and periodontal disease. Furthermore, if SLS-producing streptococci translocate into the bloodstream, SLS-dependent cytotoxicity may be induced at the ectopic site of infection. This finding confirms that SLS-producing streptococci can establish ectopic infections, explaining the clinical perspective of ectopic infections by oral streptococci in humans.

In the present study, it was shown that the SLS secreted by the β-hemolytic *S. anginosus* subsp. *anginosus* strain NCTC10713^T^ not only exhibited cytotoxicity against HSC-2 cells (Fig. 1) but also greatly induced the expression of IEGs encoding transcription factors and genes encoding inflammatory cytokines in a Ca^2+^-dependent manner (Figs. 4, 5, 6). The Ca^2+^-dependent expression of IEGs has been reported previously^23^, and the influx of extracellular Ca^2+^ is thought to be an important event that triggers increased expression of IEGs. IEGs are general terms for genes that represent rapid and transient but protein synthesis-independent increase in expression in response to extracellular signals such as growth factors and neurotransmitters^24^. Generally, although IEGs are mainly investigated in the field of neuroscience^25,26^, many studies on microbial infections have also been reported, such as infection by human pathogenic viruses^27–30^ and the pathogenicity of bacteria. Examples of the latter include bacterial pore-forming exotoxins, the relationships of IEGs to alpha-toxin produced by *Staphylococcus aureus*^31^, intermedilysin produced by *S. intermedius*^32^, and other pore-forming toxin^33^, have been reported. However, to date, there have been no reports on the relationship between IEGs and the streptococcal peptide hemolysin (SLS), also known as bacteriocin TOMMs^34^. To the best of our knowledge, this is the first report to demonstrate an SLS-dependent cellular response that induces the expression of IEGs that encode transcription factors. Ionomycin was used as a control to investigate Ca^2+^ influx into HSC-2 cells (Fig. 2). As both the secreted SLS and ionomycin induced Ca^2+^ influx into HSC-2 cells, our finding may provide the useful information for elucidating the characteristic SLS-induced cellular responses to compare the cellular responses, such as gene-expression profiles, of the HSC-2 cells treated with secreted SLS and ionomycin.

RNA-seq results of SLS-treated HSC-2 cells showed enhanced expression of 433 genes. Among these genes, we focused on IEGs encoding transcription factors, such as EGR, Fos, and Jun, and genes encoding inflammatory cytokines, such as IL-6 and CXCL8. EGRs are zinc finger transcription factors, and Fos and Jun are components of the heterodimeric transcription factor activator protein 1 (AP-1). These transcription factors regulate the transcription of various downstream genes in the cells, and the expression of these transcriptional factors have been associated with various disorders. For example, the expression of EGR1 has been associated with prostate cancer development^35^, esophageal tumor tissues^36^, and human gastric cancer progression^37^; the expression of EGR2 has been associated with the pathogenesis of fibrosis^38^ and Guillain-Barré Syndrome^39^; and the expressions of EGR3 and EGR4 has been associated with prostate cancer^40^ and small cell lung cancer^41^, respectively. In the case of AP-1, the c-Fos/JunD heterodimer is the most prevalent complex in human oral cancer tissues^42^. In addition, IL-1β-enhanced c-Fos/c-Jun heterodimer expression led to dose-dependent transcriptional activation of the collagenase promoter^43^. According to the relationship between IEG-encoding transcription factors and disorders in humans, the greatly enhanced expression of transcription factors induced by secreted SLS may trigger abnormalities in host cells and tissues. Therefore, human opportunistic AGS, which show SLS-dependent-hemolysis, should be re-evaluated as human pathogens, such as *S. pyogenes*.

Interestingly, the IEGs investigated this study are also related to disorders of the central nervous system. For example, associations have been found between EGR1 and Alzheimer's disease^44^, EGR2 and Charcot-Marie-Tooth disease^45–47^, EGR3 and schizophrenia^48–50^, and c-Jun and Krox24 (EGR1) and Alzheimer's disease^51^. In nerve cells, the expression of IEGs encoding transcription factors, such as c-Fos and EGR1, is induced by an increase in intracellular Ca^2+^ as a result of synaptic activity. This intracellular Ca^2+^-dependent increase in the expression of IEGs that encode transcription factors was also observed in HSC-2 cells treated with SLS secreted from β-hemolytic *S. anginosus* subsp. *anginosus*. HSC-2 cells are thought to regulate the expression of genes downstream of IEGs in response to membrane damage caused by SLS, and HSC-2 cells can quickly respond to infection by SLS-producing streptococci, such as by the induction of inflammatory cytokines. Judging from the results of the RNA-seq analysis, not only the expression of IEGs but also the expression of genes encoding cytokines such as IL-6 and CXCL8 increased in HSC-2 cells after incubation with the culture supernatant containing SLS (Figs. 3 and 6). Therefore, SLS-dependent initiation and/or enhancement of inflammation by SLS-producing streptococci, including β-hemolytic *S. anginosus*, may occur in vivo*.* Moreover, an association between increased expression of inflammatory cytokines and diseases such as Alzheimer’s disease and schizophrenia has been reported^52–54^. Therefore, the findings of this study are important for understanding the pathogenesis of SLS-dependent ectopic infections caused by β-hemolytic AGS. Further investigation is currently underway to elucidate the detailed mechanism of SLS-dependent pathogenicity of human habitual hemolytic oral streptococci in disorders of the host ectopically infected by these streptococci.

## Methods

### Bacterial strains and culture conditions

In this study, *S. anginosus* subsp. *anginosus* strain NCTC10713^T^ showing SLS-dependent β-hemolysis^4^ and a non-β-hemolytic mutant derived from NCTC10713^T^ without both *sagA1* and *sagA2* genes (∆*sagA*s)^4^ were used. The tested strains were preincubated in BHI broth (Becton Dickinson and Company, Franklin Lakes, NJ, USA) at 37 °C overnight with 5% CO_2_. The preincubated bacteria were inoculated into the co-cultivation medium [Eagle's minimum essential medium (EMEM, FUJIFILM Wako, Osaka, Osaka, Japan) containing 1.0% (w/v) recombinant human serum albumin (HSA; No. 19597-14, Nacalai Tesque Inc., Kyoto, Kyoto, Japan), 10% (v/v) BHI, 25 mM HEPES (pH 7.4)] to an optical density at 600 nm (OD_600_) of 0.01 and then incubated for 4 h at 37 °C in 5% CO_2_. Culture supernatants of the tested strains were obtained by centrifugation (13,000 × *g*, 5 min, RT). HSC-2 cells were incubated with the prepared bacterial culture supernatants in the presence of antibiotics (200 U/mL penicillin G and 200 U/mL streptomycin; FUJIFILM Wako) to inhibit bacterial growth. The absence of bacterial growth during the incubation of HSC-2 cells with the prepared bacterial culture supernatant was confirmed by microscopic observation.

### Human cell line and culture condition

The human oral squamous cell carcinoma cell line HSC-2 (RCB1945; RIKEN BRC, Ibaraki, Tsukuba, Japan) was cultured in EMEM cell culture medium (FUJIFILM Wako) containing 10% (v/v) heat-inactivated fetal bovine serum (FBS) and antibiotics (100 U/mL penicillin G and 100 U/mL streptomycin; FUJIFILM Wako) under cell culture conditions (37 °C, 5% CO_2_ atmosphere).

### Cytotoxicity assay

The cytotoxicity of the bacterial culture supernatant toward HSC-2 cells was evaluated using CCK-8 (Dojindo) and CellTox™ Green Cytotoxicity Assay (Promega). For the CCK-8 assay, HSC-2 cells were inoculated at 2.0 × 10^4^ cells/0.1 mL/well in a 96-well plate, incubated overnight, and then incubated with the culture supernatant prepared from each strain for an adequate time. After washing the cells with EMEM, 90 µL of fresh culture medium and 10 µL of CCK-8 reagent were added and the cells were incubated for 1 h at 37 °C in 5% CO_2_. The absorbance at 450 nm was measured using a plate reader (Infinite^®^ 200 PRO M Nano +; TECAN, Männedorf, Zürich, Switzerland) with a reference wavelength of 600 nm. To prepare the background control for this assay, cells were treated with 0.1 N HCl and incubated for a few minutes prior to the assay. For the CellTox™ Green Cytotoxicity Assay, HSC-2 cells were inoculated at 2.0 × 10^4^ cells/0.1 mL/well in a clear-bottom 96-well black plate and incubated overnight. The culture supernatant prepared from each tested strain was added to each well (0.1 mL/well) and incubated for 2 h. The cytotoxicity of the bacterial culture supernatants was evaluated according to the manufacturer’s instructions. The fluorescence of the samples was measured using a plate reader (Infinite^®^ 200 PRO M Nano +) at excitation and emission wavelengths of 490 and 525 nm, respectively.

### Measurement of intracellular Ca^2+^ concentration

Intracellular Ca^2+^ concentration was measured using a Fura-2 Calcium Kit (Dojindo), according to the manufacturer’s instructions. HSC-2 cells were inoculated at 2.0 × 10^4^ cells/0.1 mL/well in a clear-bottom 96-well black plate and incubated overnight. Briefly, the Fura 2-AM solution prepared using the loading buffer in the kit was added to the cell culture (0.1 mL/well) and incubated for 1 h under cell culture conditions to allow the incorporation of Fura 2 dye into the cells. After washing, culture supernatant prepared from the test strain was added to each well (0.1 mL/well) and incubated for 30 min. HSC-2 cells were also treated with 0.1 µM of ionomycin as the Ca^2+^-permeabilization positive control of the assay. After incubation, the cell culture supernatant was replaced with the Recording Medium (0.1 mL/well), and fluorescence was measured using a plate reader (Infinite^®^ 200 PRO M Nano +) with an emission wavelength of 510 nm and excitation wavelengths of 340 nm and 380 nm. The fluctuation in intracellular Ca^2+^ concentration was evaluated using the 340/380 nm ratio calculated from the measurements.

### RNA-seq analysis

HSC-2 cells were inoculated at 4.0 × 10^5^ cells/2.0 mL/well in a 6-well plate and incubated overnight. After washing the cells with EMEM, 2.0 mL of the culture supernatant of the tested strain containing antibiotics (200 U/mL penicillin G and 200 U/mL streptomycin) was added to each well and incubated for 3 h. Total RNA was extracted from the cells in each well using a NucleoSpin^®^ RNA Plus kit (Takara Bio Inc., Kusatsu, Shiga, Japan) and further purified using a NucleoSpin^®^ RNA Clean-up kit (Takara Bio Inc.), according to the manufacturer’s protocol. RNA-seq analysis was performed by Macrogen, Japan (Koto-ku, Tokyo, Japan).

### Quantitative reverse transcription-polymerase chain reaction (RT-qPCR)

HSC-2 cells were seeded at a density of 4.0 × 10^5^ cells/well in a 6-well plate containing 2.0 mL of cell culture medium per well and incubated overnight. The cells were then treated with 2.0 mL/well of culture supernatant prepared from the tested strains for 2 h. To investigate the contribution of Ca^2+^ influx to the expression of the target genes, HSC-2 cells were treated with the culture supernatant containing 5 mM EGTA. After washing with EMEM, total RNA was extracted from the cells in each well using the NucleoSpin^®^ RNA Plus kit (Takara Bio Inc.) according to the manufacturer’s protocol. Reverse transcription was performed using a High-Capacity cDNA Reverse Transcription Kit with RNase inhibitor (Thermo Fisher Scientific, Waltham, MA, USA) according to the manufacturer’s instructions. An oligo (dT)_15_ primer (TaKaRa Bio Inc.) was used for the reaction instead of the random primer used in the kit. Quantitative real-time PCR was conducted on a Thermal Cycler Dice^®^ Real-Time PCR System Lite TP700 (TaKaRa Bio Inc.) using TB Green^®^ Premix Ex Taq^®^ II Tli RNase H Plus (TaKaRa Bio Inc.). The primers used are listed in Table 2. Relative quantification of target gene expression was conducted using the ΔΔCt method, using *GAPDH* as the internal control gene.

**Table 2:** Primers for RT-qPCR used in this study.

Primer name	Primer sequence (5′–3′)	Size (bp)	Tm (°C)
GAPDH_forward	GTCTTCACCACCATGGAGAAGGCT	24	55.3
GAPDH_reverse	CATGCCAGTGAGCTTCCCGTTCA	23	55.0
EGR1_forward	CCCACCATGGACAACTACCCTAA	23	53.2
EGR1_reverse	GGAAAAGCGGCCAGTATAGGTGA	23	53.2
EGR2_forward	CCCTTTGACCAGATGAACGGA	21	50.5
EGR2_reverse	CTGGATGAGGCTGTGGTTGA	20	50.0
EGR3_forward	AGCAGCGACTCGGTAGTCCA	20	52.0
EGR3_reverse	GAGTCGAAGGCGAACTTTCCCA	22	52.9
EGR4_forward	GGCCACCGGCTACCCTGGAG	20	58.2
EGR4_reverse	CTAAGATGCCCGACATGAGGTTGA	24	53.6
FOS_forward	TACTCCAGGGCTGGCGTTGT	20	52.0
FOS_reverse	TCTCCTTCAGCAGGTTGGCAATC	23	53.2
FOSB_forward	AACTGCTTCTAGAAACTCTGGCTCA	25	52.2
FOSB_reverse	GAGAAAAGACAGAGGGAGAGAGACC	25	55.5
JUN_forward	GGAGGACCGGAGACAAGTGG	20	54.1
JUN_reverse	CGCCGTGGAGAAGCCTAAGA	20	52.0
JUNB_forward	GGAAAAGAAACACGCACTTAGTCTC	25	52.2
JUNB_reverse	AACACACACAAACACAAACACGTC	24	50.2
JUND_forward	CATTCCTGTTTGTAATCCTTGGTTC	25	50.6
JUND_reverse	GGCGTAACGAGACTTTACTGAAAAC	25	52.2
IL-6_forward	ATGAACTCCTTCTCCACAAGCGC	23	56.9
IL-6_reverse	GAAGAGCCCTCAGGCTGGACTG	22	60.3
IL-8_forward	ATGACTTCCAAGCTGGCCGTGCT	23	58.7
IL-8_reverse	TCTCAGCCCTCTTCAAAAACTTCTC	25	55.9

### Amino acid sequence identity/similarity

As β-hemolytic *S. anginosus* subsp. *anginosus* possess two *sagA* genes (*sagA1* and *sagA2*) in the *sag* operon, the target sequences for the analysis were picked out by both Nucleotide BLAST (https://blast.ncbi.nlm.nih.gov/Blast.cgi) and *Streptococcus anginosus* Nucleotide BLAST (https://blast.ncbi.nlm.nih.gov/Blast.cgi?PAGE_TYPE=BlastSearch&PROG_DEF=blastn&BLAST_PROG_DEF=megaBlast&BLAST_SPEC=MicrobialGenomes_1328&DB_GROUP=AllMG) using the nucleotide sequence from *sagA1* to *sagA2* of *S. anginosus* subsp. *anginosus* NCTC10713^T^ (GenBank ID: JN619420.1) as the query. Amino acid sequence identity/similarity was determined using the GENETYX-MAC Network Version 20.0.4 software (GENETYX Corp., Shibuya-ku, Tokyo, Japan). When required, the nucleotide sequence was translated into its amino acid sequence and used for the amino acid sequence identity/similarity assays.

### Statistics

Statistical evaluation was conducted using R software for Mac OS X (version 3.6.1; https://cran.r-project.org/bin/macosx/).

## Data Availability

All data generated or analyzed during this study are included in this published article.
